# Supplementary figures and images for: Genomic resources and toolkits for developmental study of whip spiders (Amblypygi) provide insights into arachnid genome evolution and antenniform leg patterning
Source: EvoDevo. 2020 Aug 28;11:18. doi: 10.1186/s13227-020-00163-w (PMC7455915; doi:10.1186/s13227-020-00163-w)

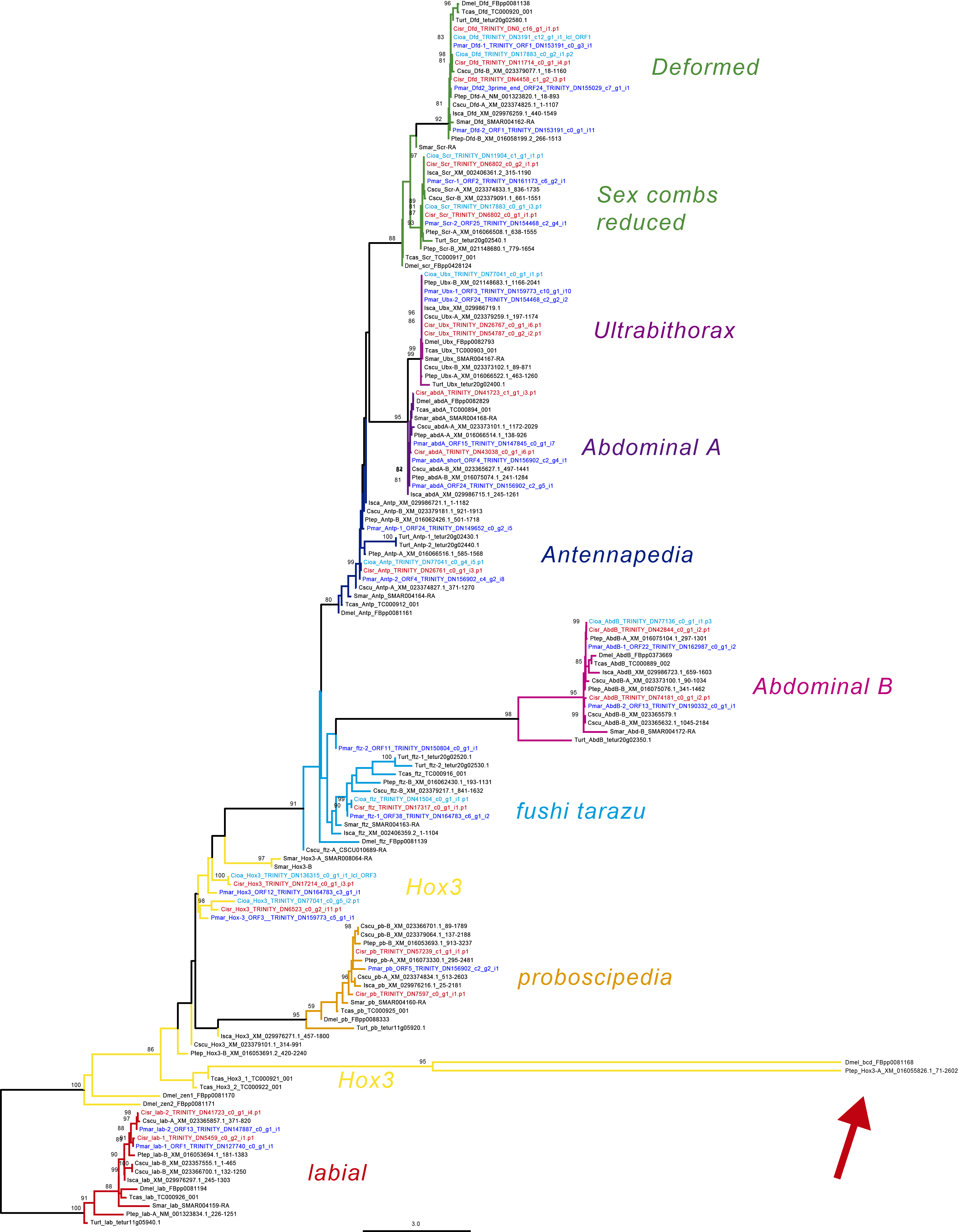

Supplement: Supplementary file 1 — Additional file 1: Fig. S1. Tree topology inferred from maximum likelihood analysis of a conserved region (75 amino acid characters) (ln L = − 4780.167) using the same terminals of Fig. 3 and including Parasteatoda tepidariorum Hox3-A paralog and Drosophila melanogaster bicoid (Hox3) (red arrow). Numbers on the notes are ultrafast bootstrap resampling frequencies (only > 80 shown). For abbreviations, see Additional file 5: Table S1. [file 13227_2020_163_MOESM1_ESM.jpg]

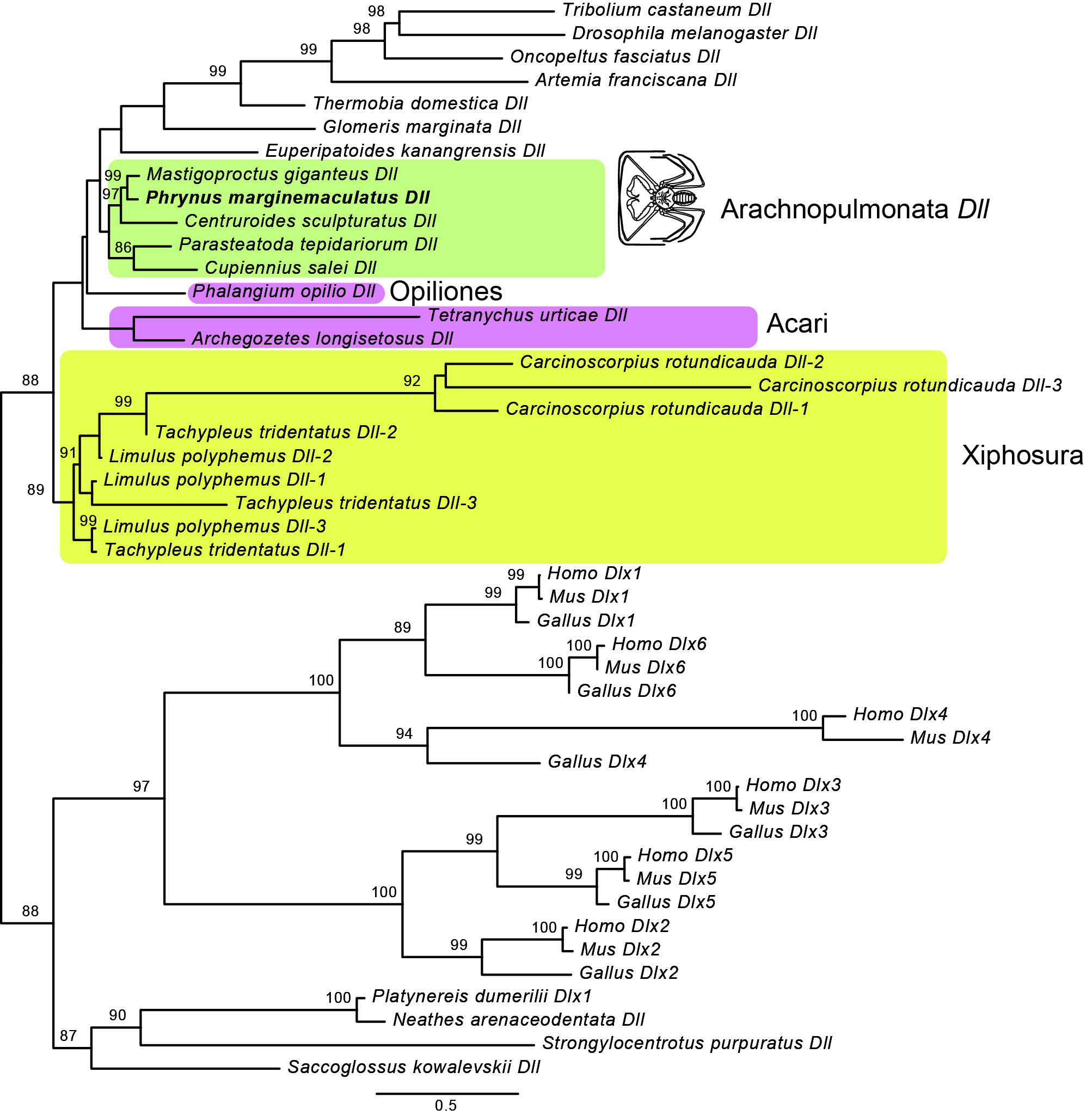

Supplement: Supplementary file 2 — Additional file 2: Fig. S2. Tree topology of Distal-less (Dll) inferred from maximum likelihood analysis of amino acid sequences (ln L = − 15,272.780). Numbers on the notes are ultrafast bootstrap resampling frequencies (only > 80 shown). Accession numbers are available in Additional file 5: Table S1. The terminal for the whip spider Phrynus marginemaculatus Dll ortholog is in boldface. [file 13227_2020_163_MOESM2_ESM.jpg]

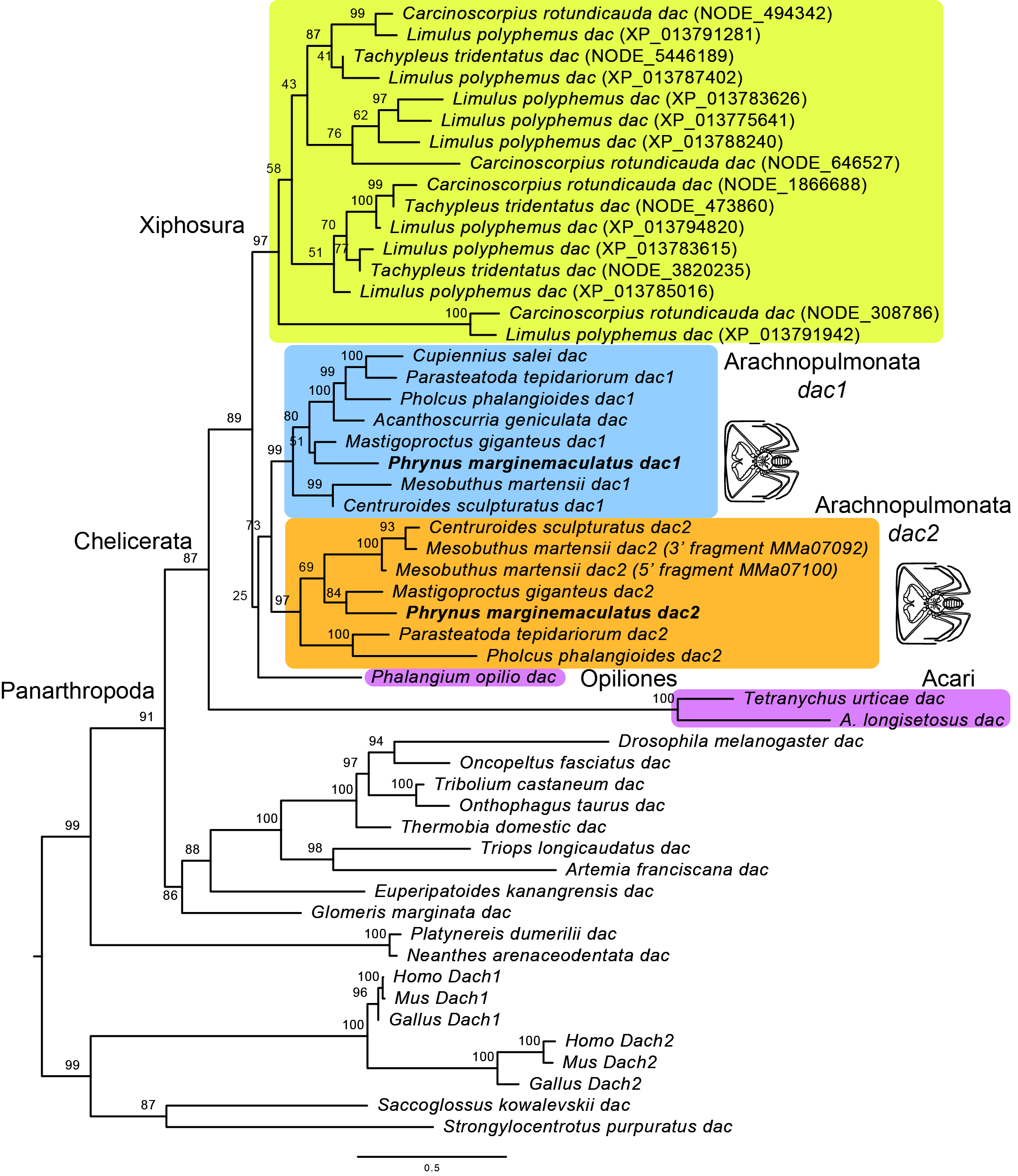

Supplement: Supplementary file 3 — Additional file 3: Fig. S3. Tree topology of dachshund (dac) inferred from maximum likelihood analysis of amino acid sequences (ln L = − 21,699.907). Numbers on the notes are ultrafast bootstrap resampling frequencies (only > 80 shown). The terminals for the whip spider Phrynus marginemaculatus dac orthologs are in boldface. The original protein alignment of all terminals (except P. marginemaculatus) is from Nolan et al. [75]. [file 13227_2020_163_MOESM3_ESM.jpg]

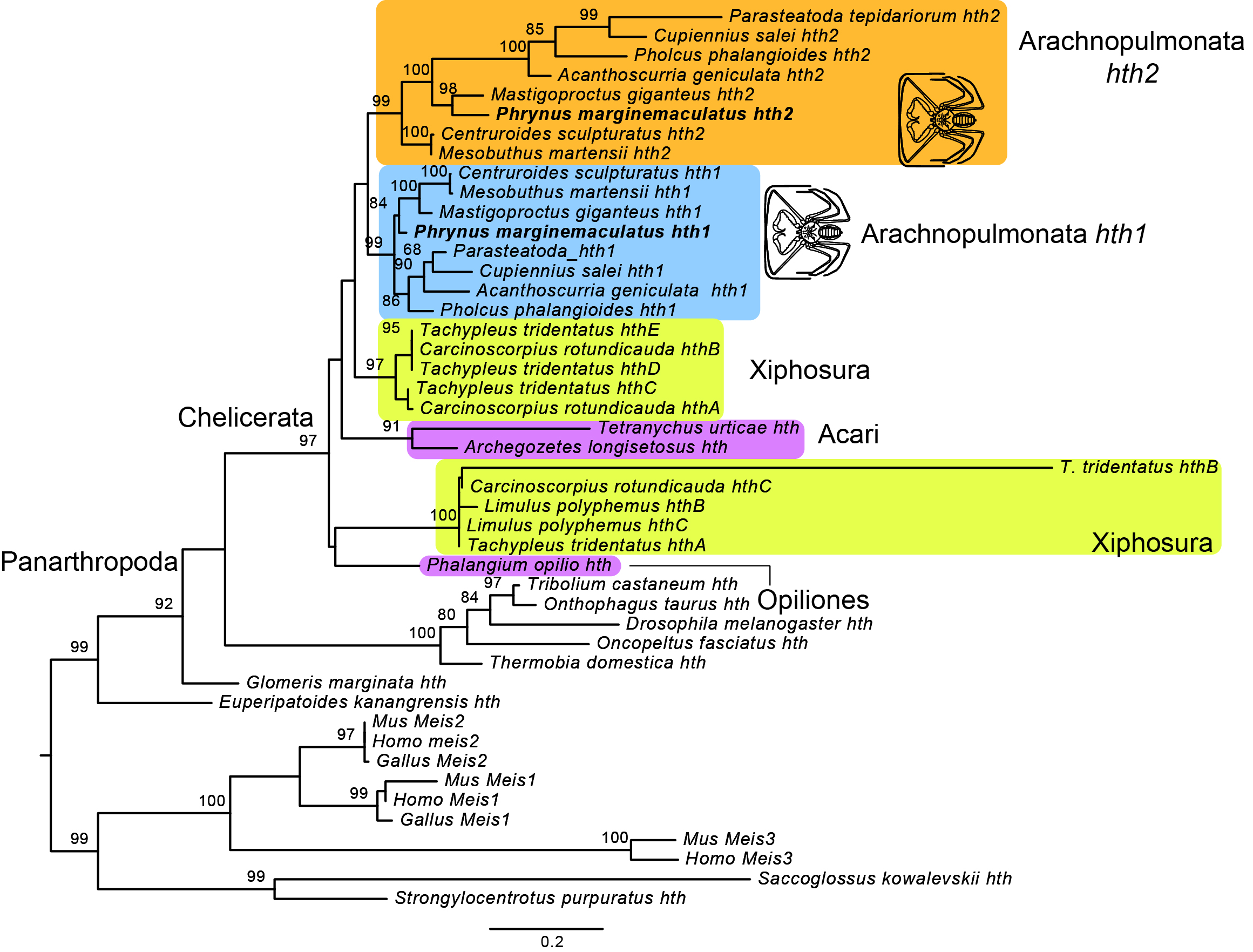

Supplement: Supplementary file 4 — Additional file 4: Fig. S4. Tree topology of homothorax (hth) inferred from maximum likelihood analysis of amino acid sequences (ln L = − 10,459.647). Numbers on the notes are ultrafast bootstrap resampling frequencies (only > 80 shown). The terminals for the whip spider Phrynus marginemaculatus hth orthologs are in boldface. The original protein alignment of all terminals (except P. marginemaculatus) is from Nolan et al. [75]. [file 13227_2020_163_MOESM4_ESM.jpg]
